# Freeze–thaw characterization process to minimize aggregation and enable drug product manufacturing of protein based therapeutics

**DOI:** 10.1038/s41598-021-90772-9

**Published:** 2021-05-31

**Authors:** Keethkumar Jain, Nazila Salamat-Miller, Katherine Taylor

**Affiliations:** Takeda Pharmaceutical Company Limited, 200 Shire Way, Lexington, MA 02421 USA

**Keywords:** Biologics, Antibody therapy, Proteins, Chemistry, Chemical biology, Pharmaceutics

## Abstract

Physical instabilities of proteins in the form of protein aggregation continue to be a major challenge in the development of protein drug candidates. Aggregation can occur during different stages of product lifecycle such as freeze–thaw, manufacturing, shipping, and storage, and can potentially delay commercialization of candidates. A lack of clear understanding of the underlying mechanism(s) behind protein aggregation and the potential immunogenic reactions renders the presence of aggregates in biotherapeutic products undesirable. Understanding and minimizing aggregation can potentially reduce immunogenic responses and make protein therapeutics safer. Therefore, it is imperative to identify, understand, and control aggregation during early formulation development and develop reliable and orthogonal analytical methodologies to detect and monitor levels of aggregation. Freezing and thawing are typical steps involved in the manufacturing of drug product and could result in complex physical and chemical changes, which in turn could potentially cause protein aggregation. This study provides a systematic approach in understanding and selecting the ideal freeze–thaw conditions for manufacturing of protein-based therapeutics. It identifies the importance of balancing different excipients with an overall goal of sufficiently reducing or eliminating aggregation and developing a stable and scalable formulation. The results demonstrated that the freeze–thaw damage of mAb-1 in aqueous solutions was significantly reduced by identification of optimal freeze–thaw conditions using first a small-scale model with subsequent at-scale verifications. The work provides a framework for successful transfer of drug product manufacturing process from small-scale to the manufacturing scale production environment especially for molecules that are susceptible to freeze–thaw induced degradations.

## Introduction

Protein therapeutics serve as powerful tools to provide treatment for several diseases. As the number of therapeutic protein products increases, protein stability has gained significant importance. Protein aggregation, one of the most common examples of protein instabilities is observed at all stages of drug development and presents major challenges to successful progression of drug candidates. The structure of a protein may change under certain conditions and lead to aggregation, which is a major event of physical instability. Presence of any aggregation in a protein pharmaceutical can impact activity, solubility, and may even present protein specific immune responses, and is generally not acceptable for product release^2,3^.

Proteins are commonly exposed to the freeze–thaw (F/T) events during bulk drug substance handling, manufacturing, and storage, as well as potential excursions during shipping^4^. Changes to the protein conformation such as partial unfolding of protein molecules during F/T has been found to result in protein aggregation^1,5^. Freezing and thawing stresses such as formation of ice-water interfaces, buffer-induced pH change, re-distribution / concentration of solutes, and phase separation can lead to complex changes in the buffer environment and result in protein aggregation^6,7^. While the mechanisms behind protein stabilization (or destabilization) by buffers are complex and not well understood, a protein’s solution environment properties such as salt type, buffers, and their concentrations have been shown to exert a strong influence on protein aggregation^8,9^.

There is an interest in the biopharmaceutical industry in developing a F/T characterization process that is systematic, prioritizes process understanding and control, and allows to evaluate the potential impact of manufacturing scale conditions on protein stability. Often passive freezing and thawing techniques are implemented during the manufacturing of monoclonal antibodies without gaining product and process specific knowledge. Such non-optimized approach can negatively impact the physical and chemical properties of protein solutions during F/T events resulting in increased overall development costs and time. With consideration of this and limited material availability at early stages of a program, in the present study, the rate combinations of *slow freeze- fast thaw and fast freeze- slow thaw* was systematically examined; depending on the type and lifecycle phase of the molecule, such information may be required early on when large amounts of material are generally not available. While the formulation excipients had been previously identified based on extensive (pre)formulation screening studies of mAb-1, the objective of this study was to present a F/T characterization approach by systematically evaluating the effects of process and formulation variables that contribute to aggregation of mAb-1, and develop a scalable process for transfer to an off-site fill/finish facility.

## Materials and methods

### Materials

The protein evaluated in these studies was a mAb-1 fusion enzyme (Fc portion of a mAb fused with an enzyme), expressed and purified at Takeda Pharmaceutical Company, Lexington, MA. The protein solutions at pH 6.0 were formulated in different concentrations of sodium phosphate, sodium chloride, and surfactant. The buffer and excipients selected for mAb-1 formulation were based on extensive (pre)formulation screening studies conducted previously. Sodium chloride (NaCl), monobasic sodium phosphate monohydrate (NaH_2_PO_4_.H_2_O), dibasic sodium phosphate heptahydrate (Na_2_HPO_4_.7H_2_O_._), and surfactant were purchased from J.T. Baker. Double deionized water was used to prepare all buffer solutions unless otherwise indicated.

### Low temperature thermal analysis

The physiochemical behavior of the mAb-1 at low temperatures was characterized by electrical resistance, observations under a freeze-drying microscope, and low temperature differential scanning calorimetry (LT-DSC)^10^. Analysis of the samples was conducted at Lyophilization Technology (Ivyland, PA). The intent of this analysis was to determine the minimum temperature necessary for complete solidification during freezing and the product’s thawing characteristics.

Resistance was measured on the protein sample in a glass sample tube using 0 to 20 megaohm resistance instrument and a ceramic resistance probe^10^. Temperature was measured using a 32-gauge thermocouple located at the ceramic probe on the opposite sides of the gold plates. The protein material was cooled and warmed at an average controlled rate of 0.5 °C per minute to assess its thermal characteristics and establish a material- specific phase transition. The resistance sample was analysed at atmospheric conditions and a deviation in resistance was used to determine an onset of the phase transition upon warming^10^. Temperature measurements were recorded every ten seconds during the analysis.

For freeze-drying microscopy, protein sample was dispensed into a glass cell and placed on a temperature-controlled freeze-drying stage. The sample cell was outfitted with two 32-gauge thermocouples placed directly into the material at the bottom and center of the cell to monitor sample temperatures. The liquid protein sample was cooled at a controlled rate of 0.5 °C per minute to a target setpoint of − 60 °C. Upon the completion of freezing, the stage was then warmed at an average controlled rate of 0.5 °C per minute. Sample behavior was observed using a Microscope capable of magnification from 16 to 330 × coupled to a camera^10^. Temperature measurements were recorded every ten seconds during the complete analysis.

LT-DSC from TA Instruments (Q200 DSC) was utilized as a means of assessing the physico-chemical behavior during freezing and warming for determining phase transitions^10^. A range of 20.0 to 21.1 mg of solution was placed in a sample pan and with a lid crimped in place. Nitrogen was used to purge the sample continuously at a flow rate of 50 mL/minute. The liquid protein sample was cooled to a temperature of − 65 °C to complete freezing and warmed at a controlled rate of 10 °C per minute. During cooling and warming, evolution or uptake of heat for the sample was monitored and the differences in heat energies were recorded^10^.

### Determination of effect of freezing and thawing rates

Studies were conducted to evaluate the effect of rate of small scale freezing and thawing on the stability of the mAb-1 formulated at 5.5 mg/mL in sodium phosphate, sodium chloride, and surfactant at pH 6.0. At a production scale, it is difficult to control the freezing and thawing rates of proteins and hence when evaluating the effect of F/T in scale down models, it is critical that the effect of different rates be incorporated in the study design. Using a small scale controlled-rate freezer (Tenney, TUJR), samples were frozen and thawed. Small scale F/T studies included either three cycles of freezing to − 50 °C and thawing to 25 °C at a fast freeze and slow thaw rates or one cycle at a slow freeze and fast thaw rates. A liquid control in a same formulation was stored at 5 ± 3 °C for the duration of the study. Samples from each test condition were taken after F/T cycles and the presence of aggregation was measured before and after each F/T by size exclusion HPLC (SE-HPLC). The following F/T programs were used:

*Slow Freeze-Fast Thaw* Freeze from 5 to − 50 °C at 0.03 °C/min., hold at − 50 °C for 2 h, thaw at 1 °C / min. to 5 °C and hold at 5 °C until analysis.

*Fast Freeze-Slow Thaw* Freeze to − 50 °C at 1 °C/min., hold at − 50 °C for 2 h, thaw at 0.03 °C / min. to − 25 °C, hold at − 25 °C for 24 h, ramp to 5 °C at 0.03 °C / min and hold at 5 °C until analysis.

For further analysis of the thawing conditions at different scales, mAb-1 drug substance at higher fill volumes (~ 20 mL, 250 mL, and 500 mL) was frozen to ≤ − 50 °C for a minimum of 48 h and subjected to rapid thawing in a circulating water bath set at either 25 ± 2 °C or 37 ± 2 °C with intermittent mixing. At the end of each F/T cycles, samples were analysed by SE-HPLC.

Additionally, mAb-1 formulated at 5.5 mg/mL in sodium phosphate, sodium chloride, and surfactant at pH 6.0 were also subjected to small scale 1 × and 3 × fast freeze and slow thaw cycles. The presence of aggregation was monitored before and after F/T by SE-HPLC and Analytical Ultracentrifugation (AUC).

### Screening of mAb-1 formulation matrices to minimize F/T induced aggregation

To determine the effect of protein concentration, salt, and sodium phosphate buffer on the F/T induced aggregation, several studies were conducted. Table 1 provides a summary of the formulation matrices that were evaluated. For all these studies, the mAb-1 formulated at pH 6.0 was subjected to multiple fast freeze and slow thawing cycles.

**Table 1:** Screening of mAb-1 formulation matrix.

Parameter	Formulation Matrix Attribute	Conditions/Ranges Evaluated
Protein	mAb-1	5 mg/mL and 15 mg/mL
Salt	Sodium Chloride	50 and 140 mM sodium chloride
Buffer	Sodium Phosphate	5 and 20 mM sodium phosphate

### Effect of phosphate and sodium chloride concentrations on freeze–thaw induced aggregation

The mAb-1’s F/T stability was examined by varying salt and phosphate buffer concentrations (Table 1). The presence of aggregation was measured before and after F/T by size exclusion HPLC.

### Effect of protein concentration

The mAb-1’s susceptibility to F/T induced aggregation was examined at 5 mg/mL and 15 mg/mL (Table 1).

### Determination of free Thiol(s) in mAb-1 drug substance

Cysteine residues in a protein may exist as free thiols (exposed or buried) and may be involved in the formation of disulfide bonds. The presence of disulfide bonds has been known to provide conformational stability to native protein structure, however in their reduced form (free thiols), they may contribute to the instability of the molecule (intra- and intermolecular disulfide bond formation) leading to covalent protein aggregation. Literature confirms that formation of intermolecular disulfide bonds promotes protein aggregation in multiple ways^1,11–14^. To that effect, studies were conducted to quantitatively determine if any free thiols were present in mAb-1.

The free thiol assay was based on the reaction of free thiols with the Ellman’s reagent, [5,5ʹ-dithiobis (2-nitrobenzoic acid), or DTNB] to form a colored thiolate anion (TNB^−2^), quantitated spectrophotometrically at a wavelength of 412 nm. Guanidine-hydrochloride denatured samples were incubated in a 96-well microtiter plate with the Ellman’s reagent at ambient temperature and the absorbance of the solution in each well was measured at 412 nm. The measured absorbance was converted to molar concentration of free thiol using a standard curve of L-cysteine hydrochloride treated similarly in the denaturing buffer. The molar ratio of free thiol to the protein was then reported.

## Results

### Low temperature thermal analysis

**Figure 1:**
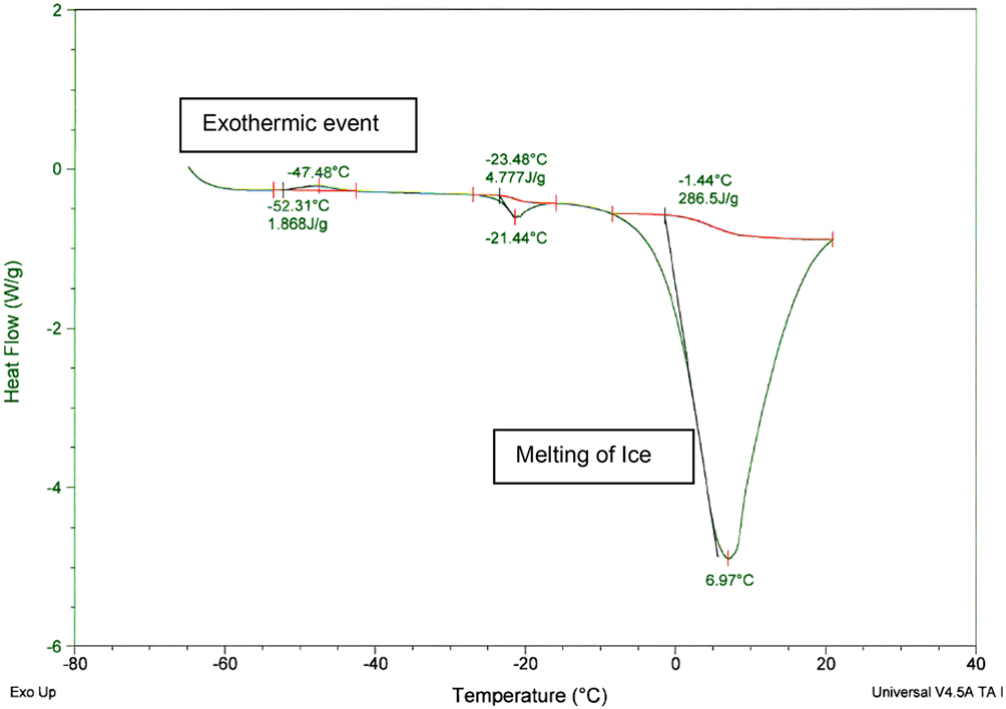
Low temperature DSC profile of mAb-1.

The phase transition temperature acquired from the resistance graph suggests that cooling to temperatures of at least − 31 °C would be required for achieving solidification. Observations under the microscope showed that the sample matrix appeared completely solidified at a temperature of − 40 °C, first exhibiting signs of melting at − 28 °C. Low temperature DSC results indicated that an exothermic event occurred at a peak of approximately – 48 °C (Fig. 1). It was thus recommended that the mAb-1 be cooled to temperatures below – 48 °C to ensure complete solidification.

### Determination of effect of freezing and thawing rates

To the best of the authors’ knowledge, there is no published evidence on standards and consistently used freezing and thawing rates adopted across the industry. Given that F/T rates can affect the stability of proteins and it may not always be possible to control them during the drug product manufacturing, these specific F/T rate combinations were evaluated first at small scale in order to identify any molecular susceptibility and define the optimal operating conditions for successful drug product manufacturing. It was identified that mAb-1 was susceptible to F/T stress and significant aggregation was observed when subjected to fast freezing and slow thawing as compared to slow freeze and fast thaw. The extent of aggregation increased multifold after additional cycles of fast freeze and slow thaw (Table 2). Based on our extensive understanding of the molecule, it was hypothesized that it is most likely that slow thawing is contributing to aggregate formation.

**Table 2:** Effect of freezing and thawing rates on the freeze–thaw stability of mAb-1 formulated at pH 6.0.

F/T Conditions	Number of cycles	Fill volume	Refrigerated control (% Aggregates)	Post F/T (% Aggregates)
Slow freeze-fast thaw	1x	1 mL in 2 mL Vial	0.1	0.4
Fast freeze-slow thaw	1x	1 mL in 2 mL Vial	0.2	3.2
	2x			8.4
	3x			14.4

Once the molecular susceptibility was identified and fast thawing was determined to be ideal, a water bath set at either 25 ± 2 °C or 37 ± 2 °C provided an option for this case, as compared to conventional thawing of proteins either at controlled room temperature conditions (20 ± 5 °C) or refrigerated (5 ± 3 °C). Additional F/T studies as outlined in Table 3 at different fill volumes from 20 to 500 mL in various appropriately sized containers were also evaluated and no significant increase in percent aggregates was detected.

**Table 3:** Evaluation of freeze–thaw stability of mAb-1 after fast and controlled thawing (water bath).

F/T conditions	Number of cycles	Fill volume	Refrigerated control (% Aggregates)	Post F/T (% Aggregates)
Freeze to ≤ − 50 °C for ≥ 48 h; Thaw in 25 ± 2 °C water bath	1 ×	20 mL in 60 mL PETG Bottle	0.1	0.1
Freeze to ≤ − 50 °C for ≥ 48 h; Thaw in 37 ± 2 °C water bath	1 ×	20 mL in 60 mL PETG Bottle	0.1	0.1
Freeze to ≤ − 50 °C for ≥ 48 h; Thaw in 25 ± 2 °C water bath	1 ×	250 mL in 500 mL PETG Bottle	0.7	0.8
	2 ×			0.9
Freeze to ≤ − 50 °C for ≥ 48 h; Thaw in 37 ± 2 °C water bath	1 ×	250 mL in 500 mL PETG Bottle	0.7	0.8
	2 ×			0.9
Freeze to ≤ − 50 °C for ≥ 48 h; Thaw in 25 ± 2 °C water bath	1 ×	500 mL in 1L PC Bottle	0.6	0.7

*F/T* Freeze–thaw, *PC* Polycarbonate, *PETG* polyethylene terephthalate glycol.

Since the fast freeze and slow thaw model generated significant level of aggregates, the model was also utilized to compare the stability-indicating capabilities of SE-HPLC and analytical ultracentrifugation (AUC) to detect aggregates of mAb-1. Varying levels of aggregates were prepared by fast freezing and slow thawing of mAb-1 solutions and tested by SE-HPLC and AUC. A strong correlation (r^2^ > 0.95) was observed in the capabilities of both the methods to quantify aggregates (Table 4). The results indicated that the quantification of aggregate levels by SE-HPLC is accurate and that any dilution of protein sample during sample preparation and analysis by HPLC does not result in underestimation of the aggregate levels in the protein.

**Table 4:** Comparison of percent aggregates quantified by size exclusion HPLC and analytical ultracentrifugation.

F/T Conditions	% Aggregates by SE-HPLC	% Aggregates by AUC
Baseline	0.1	0.1
Fast Freeze-Slow Thaw (1x)	4.3	6.1
Fast Freeze-Slow Thaw (3x)	12.9	11.5

In order to evaluate the effect of more rapid thawing on aggregation of mAb-1, a circulating water-bath set at 25 ± 2 °C and 37 ± 2 °C was employed to facilitate heat exchange during the thaw process. Results from the additional analysis of the fast and controlled thawing conditions (water bath set at either 25 ± 2 °C or 37 ± 2 °C) of frozen mAb-1 drug substance at different fill volumes ranging from 20 to 500 mL are also summarized in Table 3. No significant increase in aggregation was detected when mAb-1 was thawed at either temperatures.

### Effect of phosphate and sodium chloride concentrations on freeze–thaw induced aggregation

**Figure 2:**
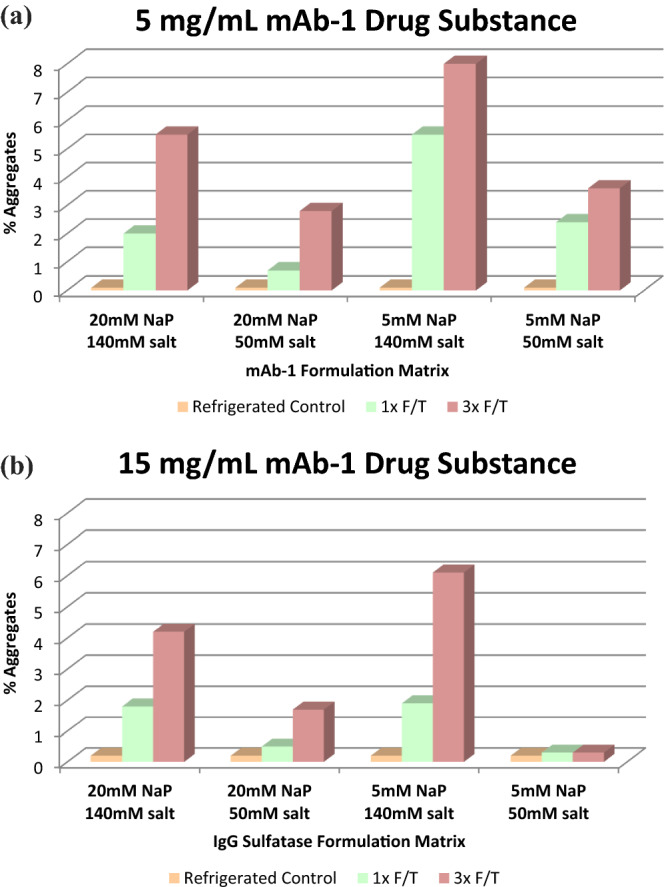
Freeze–Thaw Results for mAb-1 Solution at (**a**) 5 mg/mL and (**b**) 15 mg/mL. *NaP* = sodium phosphate, *salt* = sodium chloride.

In the presence of sodium phosphate, formulations with isotonic amounts of sodium chloride had an increased level of high molecular weight species after F/T stress. Formulations containing 50 mM sodium chloride had lower amounts of aggregation. Formulation with a lower phosphate concentration and high sodium chloride concentration demonstrated the highest level of aggregates post F/T stress (Fig. 2). Overall, formulations with reduced sodium chloride concentrations demonstrated the least susceptibility towards the F/T stress.

### Effect of protein concentration

The mAb-1 at higher concentration (15 mg/mL) demonstrated enhanced stability towards F/T stress as compared to mAb-1 at 5 mg/mL. Based on the results, the recommended formulation matrix should include high protein concentration and reduced sodium chloride.

The overall impact of F/T process parameters (number of cycles, salt and buffer concentration, and protein concentration) on protein aggregation, as generated from the small-scale studies, is summarized in Fig. 2.

### Determination of free Thiol(s) in mAb-1 drug substance

The free thiol amount was quantitatively determined in few mAb-1 formulations and the data are summarized in Table 5. Based on the analysis, free thiols were present.

**Table 5:** Quantitative determination of free thiols in mAb-1 protein formulations.

Sample name	Free thiol to protein molar ratio
mAb-1 at 15 mg/mL in 20 mM Sodium Phosphate and 140 mM salt	1.5
mAb-1 at 15 mg/mL in 20 mM Sodium Phosphate and 50 mM salt	1.6
mAb-1 at 5 mg/mL in 20 mM Sodium Phosphate and 140 mM salt	1.6

### Confirmation study

A thorough understanding of the impact of freezing and thawing processes is critical during storage and manufacturing of biopharmaceuticals. The small scale F/T studies demonstrated that mAb-1 was susceptible to freeze–thaw stress and that the critical factor responsible for F/T induced aggregation of mAb-1 was the thaw time. A F/T characterization approach using information gained from small-scale F/T models at the development stage (Tables 2 and 3) and evaluation of the protective/destabilizing effect of previously selected formulation components helped in sufficiently minimizing and eliminating aggregation.

**Figure 3:**
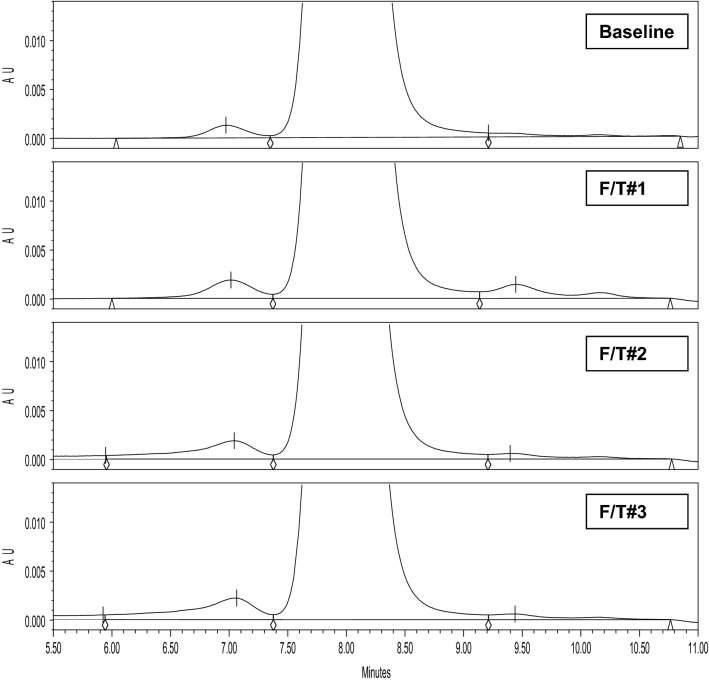
Overlay of SE-HPLC Profiles for mAb-1 Drug Substance (high protein concentration and reduced salt) Post Freezing and Water Bath Thawing at 25 ± 2 °C.

Although small scale models provided an insight into the impact of F/T process parameters on protein stability, F/T studies at an at-scale fill volume, with the optimized formulation and fast thawing conditions using water bath, were needed to confirm the small scale results at scale. With the knowledge gained from the small scale models, further studies were conducted to accurately simulate the effect of freezing and thawing at the manufacturing scale. The goal of the latter was to confirm the final thaw condition and to avoid unwanted complications during drug substance thawing for drug product manufacturing^15,16^. To confirm the selected conditions, the mAb-1 drug substance (15 mg/mL) in the proposed low phosphate concentration, reduced sodium chloride, surfactant formulation matrix and at an at-scale fill volume of 1L in 2L polycarbonate bottles were frozen at ≤ − 65 °C and thawed in a circulating water bath set at 25 ± 2 °C for multiple F/T cycles prior to analysis. As shown in Table 6, repeated F/T cycles did not result in the development of aggregates, as detected by SE-HPLC, and no impact was observed to other product quality attributes. Representative SE-HPLC profiles from the study are shown in Fig. 3.

**Table 6:** Results from at-scale freeze/thaw of mAb-1.

Sample	Appearance	pH	SE-HPLC		CE-SDS		Potency (U/mg)	Protein Concentration (mg/mL)
			% Main Peak	% HMW	Reduced (%)	Non-reduced (%)		
**Baseline**	Conforms	6.0	> 99	0.2	> 98	> 98	> 50	~ 15
**F/T # 1**	Conforms	6.0	> 99	0.2	> 98	> 98	> 50	~ 15
**F/T # 2**	Conforms	6.1	> 99	0.2	> 98	> 98	> 50	~ 15
**F/T # 3**	Conforms	6.1	> 99	0.3	> 98	> 98	> 50	~ 15

Conforms = Colorless, slightly opalescent, and does not contain any particles.

### mAb-1 stability studies

The stability data available to-date demonstrate that the high protein concentration and reduced sodium chloride formulation continues to meet the target criteria for the quality attributes tested at the long-term storage conditions (Table 7). No significant changes in the purity of the mAb-1 has been observed by chromatographic and electrophoretic methods of SE-HPLC, CE-SDS, and peptide mapping over time when stored at the intended long-term storage condition. The structural integrity of the glycans, the potency, appearance, pH, and content of mAb-1 were also maintained.

**Table 7:** Long-term stability data for one representative lot of the mAb-1 drug substance.

Test	Month					
	Baseline	3	6	9	12	18
Appearance	Conforms	Conforms	Conforms	Conforms	Conforms	Conforms
SE-HPLC (% main peak)	> 99%	> 99%	> 99%	> 99%	> 99%	> 99%
SE-HPLC (% HMW)	< 0.5%	< 0.5%	< 0.5%	< 0.5%	< 0.5%	< 0.5%
Potency (U/mg)	> 50	> 50	> 50	> 50	> 50	> 50
pH	6.0	6.1	6.1	6.0	6.1	6.0
Protein Concentration (mg/mL)	~ 15	~ 15	~ 15	~ 15	~ 15	~ 15
CE-SDS (Reduced)	> 98%	> 98%	> 98%	> 98%	> 98%	> 98%
CE-SDS (Non-reduced)	> 98%	> 98%	> 98%	> 98%	> 98%	> 98%

Conforms = Colorless, slightly opalescent, and does not contain any particles.

## Discussion

Initial formulation development studies demonstrated that mAb-1 was susceptible to F/T stress resulting in soluble aggregate formation. The aggregates are likely the result of protein unfolding during freezing and subsequent association of unfolded molecules via covalent linkages. mAb-1 contains a minimum of one free thiol that is unexposed in its native state, but upon denaturation has the potential to facilitate intermolecular thiol exchange. Aggregates may also form by non-covalent association of partially unfolded species. It is likely that due to the transient nature of the unfolded species, time is a critical parameter in determining the extent of aggregation, irrespective of the mechanism of association. As the time of thaw is extended, instances of protein–protein contacts between unfolded molecules and aggregates increase, resulting in greater percentage of high molecular weight mAb-1 aggregates. While, it is possible that both fast freezing and slow thawing could be contributing to aggregation in the case of mAb-1, it is most likely that slow thawing is causing more aggregate formation based on our extensive understanding of the molecule. The at-scale mAb-1 drug substance bottles are frozen in large freezers, which is unlikely to be a fast freeze, and are thawed using a water bath. Overall, the results support our hypothesis that the critical factor responsible for F/T induced aggregation of mAb-1 is the time of thaw and that slow thawing has a negative impact on the F/T stability of the protein.

At the manufacturing scale, thawing using a water bath set at either 25 ± 2 °C or 37 ± 2 °C provides an option for fast thawing as compared to conventional thawing of proteins at either controlled room temperature conditions (20 ± 5 °C) or refrigerated (5 ± 3 °C) thawing. Based on the study results and molecular sensitivity to thaw, the controlled thawing conditions with intermittent mixing of bottles to ensure homogenization have been implemented at the contract fill site to maintain the quality of mAb-1 upon freeze–thaw.

The data in this study suggested that the mAb-1 in low salt, low phosphate buffered solutions and at high protein concentration was least susceptible to aggregation by F/T stress. High protein concentration provided an enhanced stabilizing effect with overall lower level of aggregation observed in comparison to levels seen at low protein concentrations. Although contrary to the popular belief that higher protein concentration corresponds to increased likelihood of unwanted self-association and potentially increased aggregation, research evidence suggests that increasing the protein concentration, to a certain extent, can sometimes reduce the fraction of protein molecules exposed to the ice-liquid interface and thus have a positive impact on minimizing aggregation^1,5,17^. In this study, as the concentration of salt in phosphate buffered saline solutions was increased, the amount of protein aggregation increased. Such high electrolyte concentrations disrupt the hydration shell around protein molecules by causing water around them to move into the bulk solution and thereby exposing the hydrophobic regions. All these changes have the potential to create a hostile environment for the protein and would be expected to disrupt the delicate balance of forces that maintains a protein in its native conformation. As the protein is being frozen, salt may increase protein–protein interactions and lead to aggregation; therefore, more aggregation would therefore be expected at higher salt concentrations^18^. Some proteins are susceptible to spontaneous unfolding at cold temperatures experienced during F/T cycles, which may be attributed to weakening of hydrophobic forces^19,20^, or even potentially forming new covalent bonds (such as when free buried thiols are at least partially exposed), at reduced temperatures. The fact that a salt effect is exaggerated at lower phosphate solutions suggests a pH and a strong ionic strength component to aggregation^21^. As a solution freezes, the pH of a phosphate buffered solution is expected to drift due to the differential solubility of the phosphate salts and the selective precipitation of the disodium phosphate^22^. At low phosphate and protein (5 mg/mL) concentrations, there may not be sufficient buffering capacity to prevent a pH-induced aggregation. At high protein concentrations with same amount of phosphate, however, no buffer effect was observed suggesting that either the protein had adequate buffering capacity or high protein concentration provided cryoprotection to prevent pH changes.

## Conclusion

The results of this study demonstrate that the fusion mAb-1 is susceptible to F/T induced degradation and identified the risk of F/T parameters on aggregation. With the use of small-scale models and confirmatory at-scale assessments, an optimal F/T process was defined for mAb-1 to be implemented at the manufacturing scale. The results also suggested that our fusion protein, in phosphate buffer solution, should be thawed rapidly to maximize heat transfer and minimize the risk of aggregate formation due to prolonged exposure to high solute concentrations and exposure of buried amino acids; both were mitigated by reducing the concentration of sodium chloride in the drug substance formulation and increasing the protein concentration. The stability of the high concentration and reduced salt drug substance formulation has been confirmed with multiple F/T cycles and good long-term stability to-date. It should be noted that our results apply only to our specific mAb-1 formulation and other protein formulations may behave differently, which necessitate additional case-by-case evaluation and understanding. However, it is expected that general governing molecular behavior is universal: protein damage by concentrated salt solution and / or exposure of undesirable hidden amino acids with thiol groups during a slow thawing process. Therefore, it is recommended to evaluate and consider fast thawing for manufacturing of protein-based therapeutics. While the manuscript provides an insight into different controlled thawing conditions, it should be noted that an optimal freezing condition also depends on the protein’s solution conditions and should be determined by further studies. The findings of this manuscript may drive process development and formulation development scientists for the selection of appropriate handling conditions for at-scale manufacturing of protein-based therapeutics to avoid unwanted complications during tech transfer.

## Data Availability

Data generated or analyzed during this study and not included in this manuscript can be shared with the Editorial Board Members and referees on reasonable request to the corresponding author.
